# Featured Papers in Computer Methods in Biomedicine

**DOI:** 10.3390/bioengineering11100985

**Published:** 2024-09-29

**Authors:** Luca Mesin

**Affiliations:** Mathematical Biology and Physiology, Department of Electronics and Telecommunications, Politecnico di Torino, 10129 Turin, Italy; luca.mesin@polito.it; Tel.: +39-0110904085

## 1. Introduction

Recent years have seen progress in the intersection of computer science and biomedicine, progress that has led to significant advancements in healthcare diagnostics, treatment, and patient care. The application of machine learning (ML) and computational modeling in biomedicine is revolutionizing the way various clinical conditions are understood, diagnosed, and treated. This Special Issue presents a collection of seven articles that showcase the latest research and technological innovations in this interdisciplinary field. These papers highlight new approaches and methodologies that promise to enhance medical outcomes and improve the quality of patient care.

## 2. This Special Issue

This Special Issue consists of seven papers covering a few topics within a wide range of research fields of interest.

In order to predict low bone mineral density (BMD) in older women without the need for dual-energy X-ray absorptiometry (DXA), different ML approaches (i.e., logistic regression, decision trees, random forests, gradient boosting trees, and lightGBM) are used in [1]. They were assessed using the medical records of 2541 female patients. The model with the highest accuracy (83.4%) and area under the curve (AUC = 0.961) was the lightGBM. Three main factors that affected the prediction were age, BMI, and alanine transaminase levels. This methodology raises the possibility of non-invasive osteoporosis screening techniques and is consistent with related research endeavors centered around predictive modeling in the medical field [2,3].

Addressing the variability in microbiome data, a method is proposed in [4] to improve the generalizability of ML models for predicting different diseases, including colorectal cancer and Crohn’s disease, as well as immunotherapy response. By integrating datasets from multiple studies, the authors enhanced model performance, particularly with random forest models, through strategic data combination and feature selection. This methodology is critical in addressing the challenges of cross-study variability, as demonstrated in other works focusing on microbiome data integration and disease prediction [5,6].

In [7], a novel computational strategy is introduced to create patient-specific statistical reconstructions of healthy anatomical structures from computed tomography (CT) scans of damaged structures. Focusing on hip arthroplasty, the study demonstrates significant morphological differences between traditional prostheses and reconstructed healthy anatomy, emphasizing the potential for more accurate prosthetic designs. This approach aligns with current trends in personalized medicine and computational modeling for surgical planning and prosthetic development [8,9].

A deep learning algorithm for detecting atrial fibrillation (AF) from ECG data is introduced in [10]. By preprocessing ECG signals and employing a fine-tuned EfficientNet B0 model, the method achieved an impressive F-1 score of 88.2% and an accuracy value of 97.3%. This work underscores the growing role of artificial intelligence (AI) in cardiology, complementing other research efforts on automated arrhythmia detection [11,12,13].

Empirical mode decomposition (EMD) is used in [14] to classify Parkinson’s disease (PD) patients based on balance control data. By extracting temporal and spectral features from stabilometric signals, the authors achieved high classification accuracy (92%) through using a support vector machine (SVM) classifier. This innovative approach enhances the process for diagnosing PD, aligning with similar advances of signal processing applied to neurodegenerative diseases [15,16,17,18].

A comprehensive review of various methodologies for analyzing EEG data to understand brain connectivity is described in [19]. The authors categorize different metrics and emerging trends, such as high-order interactions and graph theory tools, providing a detailed account of the current state and future directions in EEG connectivity analysis. Functional connectivity, combined with topological indexes from graph theory, is finding many different applications, ranging from the investigation of neuronal networks in vitro with micro-electrode arrays [20] to the diagnosis of pathological conditions based on EEG data [21,22]. This work complements the existing literature on brain network analysis and connectivity [23,24].

Finally, this Special Issue also contains a review exploring the application of EEG-based brain–machine interfaces (BMI) to assist older adults, focusing on technical and user-related aspects [25]. The authors highlight the potential of BMIs to improve the quality of life of elderly individuals, particularly those with cognitive impairments. This aligns with broader research on assistive technologies and neurorehabilitation for aging populations [26]. Many recent results on the estimation of the human will from the cortical activity measured by EEG have been obtained [27,28,29] and can hopefully contribute in the future to support healthy aging.

In summary, the seven papers featured in this Special Issue showcase a variety of methodologies to advance computer methods in biomedicine. These methodologies range from ML techniques [1,4,10,14] to computational modeling and signal processing [7,19]. Two papers provide comprehensive reviews, summarizing existing methodologies and their applications in biomedicine [19,25].

## 3. Future Perspectives

This Special Issue, focusing on computer methods in biomedicine, describes significant advances in the application of computational techniques to medical scenarios. However, these studies also indicate several future directions that could be further explored:

- An important objective could be the use of enhanced ML and AI approaches to develop more personalized models [30].
- Enhancing the interpretability of AI models could ensure that they become trusted and fully understood by clinicians [31].
- The integration of multimodal data from multiple sources, such as data derived from imaging, genomics, and proteomics, as well as clinical data, is another important direction that needs to be investigated further in order to provide a comprehensive view of patient health [32].
- Real-Time data processing is fundamental to supporting timely clinical decisions [33].
- Improving algorithms for EEG and ECG analysis is important for better capturing the complexity of these physiological signals [34].
- Translation into clinical applications requires the resolution of technical issues related to the difference between research settings and clinical practice [35]. Moreover, regulatory and ethical challenges related to the use of AI and ML in healthcare should be carefully addressed [36].
- Most of the above issues can only be resolved by promoting collaboration between computer scientists, biomedical researchers, and clinicians to foster innovation.

The future of computer methods in biomedicine is promising, and such methods have the potential to revolutionize healthcare through improved data analysis, tailored treatments, and better diagnostic tools. Advancements in ML, data integration, signal processing, clinical implementation, and interdisciplinary collaboration will be crucial in driving this field forward. By addressing current limitations and exploring new frontiers, researchers can further enhance the impact of computational methods on patient outcomes and overall healthcare quality.

## 4. Conclusions

This Special Issue illustrates the potential of computer methods in biomedicine. From enhancing diagnostic accuracy with ML to developing patient-specific treatments and understanding complex brain connectivity, these studies represent significant advancements in the field. With the continued integration of computational tools in biomedical research, the future holds great promise for more personalized and effective healthcare solutions.
